# Physical activity and associated medical cost savings among at-risk older adults participating a community-based health & wellness program

**DOI:** 10.1371/journal.pone.0198239

**Published:** 2018-06-12

**Authors:** Samuel D. Towne, Yajuan Li, Shinduk Lee, Matthew Lee Smith, Gang Han, Cindy Quinn, Yuxian Du, Mark Benden, Marcia G. Ory

**Affiliations:** 1 Health Promotion & Community Health Sciences, School of Public Health, Texas A&M University, College Station, Texas, United States of America; 2 Center for Population Health and Aging, Texas A&M University, College Station, Texas, United States of America; 3 Southwest Rural Health Research Center, Texas A&M University, College Station, Texas, United States of America; 4 Department of Agricultural Economics, Texas A&M University, College Station, Texas, United States of America; 5 Department of Environmental and Occupational Health, School of Public Health, Texas A&M University, College Station, Texas, United States of America; 6 Department of Health Promotion and Behavior, College of Public Health, The University of Georgia, Athens, Georgia, United States of America; 7 Department of Epidemiology and Biostatistics, School of Public Health, Texas A&M University, College Station, Texas, United States of America; 8 Department of Health Policy and Management, School of Public Health, Texas A&M University Health Science Center, College Station, Texas, United States of America; Ospedale S. Corona, ITALY

## Abstract

**Introduction:**

Physical activity declines are seen with increasing age; however, the US CDC recommends most older adults (age 65 and older) engage in the same levels of physical activity as those 18–64 to lessen risks of injuries (e.g., falls) and slow deteriorating health. We aimed to identify whether older adults participating in a short (approx. 90-minute sessions) 20 session (approximately 10-weeks) health and wellness program delivered in a community setting saw improvements in physical activity and whether these were sustained over time.

**Methods:**

Employing a non-equivalent group design, community-dwelling older adults were purposely recruited into either an intervention or comparison group. The intervention was a multicomponent lifestyle enhancement intervention focused on healthy eating and physical activity, including structured physical activity exercises within the class sessions. Two groups were included: intervention (survey group: n = 65; accelerometer subgroup: n = 38) and the comparison group (survey group: n = 102; accelerometer subgroup: n = 55). Measurements were made at baseline and approximately three months later to reflect immediate post-treatment period (survey, accelerometer) with long-term follow-up 6 months after baseline (survey). Adults not meeting the physical activity guidelines (i.e., 150/75 minutes of moderate-to-vigorous physical activity or MVPA) were targeted for subgroup analyses. Paired t-tests were used for bivariate comparisons, while repeated measures random coefficient models (adjusting for propensity scores using inverse probability of treatment weighted (IPTW) estimation) were used for multivariate models. Estimated medical costs associated with gains in physical activity were also measured among survey respondents in the intervention group.

**Results:**

The accelerometer group contained 38 participants in the intervention group with 71% insufficiently active at baseline and 55 participants in the comparison group with 76% insufficiently active at baseline (<150 weekly MVPA minutes). The survey group contained 65 participants in the intervention group with 73.85% insufficiently active at baseline and 102 participants in the comparison group with 76.47% insufficiently active at baseline. In paired t-tests with the accelerometer group, a moderate effect size (-0.4727, p = 0.0210) indicating higher MVPA was found for intervention participants with <150 weekly MVPA at baseline. In fully adjusted analyses using propensity score matching, among the subjectively measured physical activity (survey) group, there was a differential impact from baseline to 6-month post among the intervention group with an improvement of 160 minutes among all study participants (p < .0001) versus no difference among the comparison group. For those insufficiently active at baseline, there was an improvement of 103 minutes among intervention (p < .0001) and 55 minutes among the comparison (p < .0001) with the improvement of the intervention significantly greater than that among the comparison (p = 0.0224). Further, among those insufficiently active at baseline there was a relative cost savings from baseline to 6-months over and above the estimated cost of the intervention estimated between $143 and $164 per participant.

**Discussion:**

This intervention was able to reach and retain older adults and showed significant MVPA gains and estimated medical cost savings among more at-risk individuals (baseline <150 MVPA). This intervention can be used in practice as a strategy to improve MVPA among the growing population of older community-dwelling adults.

## Introduction

In the US, the older adult population is estimated to grow from 319 million in 2014 to 417 million in 2060 [1]. It is well known that physical activity is essential for health promotion and disease prevention and lowering the risk of premature death [2], especially among older adults who typically have more chronic conditions but engage in less physical activity than their younger counterparts [3]. This confluence of population aging, growing prevalence of age-related diseases, and increased lifestyle risk factors with aging presents a timely and critical need for interventions at multiple levels. Multimodal interventions designed to increase physical activity for middle-aged and older adults are a potential public health solution for improving overall health among an aging population [4].

There are a growing number of evidence-based interventions targeted at increasing physical activity among insufficiently physically active adults [5]. In particular, behavior health interventions, while not sufficient as the sole solution for increasing physical activity at the population level, hold promise for reaching at-risk populations such as older, insufficiently physically active adults [6]. Further, several more recent interventions have targeted older individuals focusing on both clinical and behavioral outcomes such as chronic disease [7–9], falls [10, 11], physical activity [12, 13], and nutrition [14, 15]. Despite the proliferation of evidence-based programs for older adults [5], widespread dissemination and sustainability of physical activity programs for older adults has yet to be realized across the nation. Thus, there is a need for translational research exploring the evidence-base for effective physical activity-based programs that can be scaled and sustained. It is especially important to reach the growing population of older adults who are currently not meeting the Surgeon General’s recommended activity levels [16].

### Aims

We aimed to assess the physical activity outcomes of a multimodal lifestyle enhancement intervention targeted to those in the ages of 45 and older in Texas. This program, *Texercise Select*, is designed to reach at-risk community-dwelling older adults (e.g., insufficiently physically active) in need of lifestyle interventions targeting physical activity. We hypothesized that individuals exposed to the intervention would have associated gains in physical activity and that these gains would be greater among gains (if any) among the comparison group. Moreover, among the intervention group, there would be greater improvements among those insufficiently active as compared to those already meeting physical activity guidelines. Further, that estimated medical cost savings associated with these hypothesized gains in physical activity would be greater than the associated costs of the intervention, given that insufficient physical activity is associated with significant medical costs in national studies [13].

## Methods

We conducted a case/comparison quasi-experimental study to assess changes in physical activity at both short-term (immediately following the intervention or three months from entry into the study) and longer-term (6 months from entry into the study) among community-dwelling adults approximately 60 and older. Henceforth, we refer to cases as the intervention group (those exposed to the intervention) and comparisons as the comparison group (those not exposed to the intervention). *Texercise Select* is a lifestyle intervention targeting physical activity and other healthy behaviors (e.g., healthy diet). The program covers 20 educational and physical activity sessions (approximately 90-minutes in duration), twice a week for approximately 10-weeks. The program is mandated by the state to be free to participants. The program is delivered in a community setting (e.g., senior center, community center) with a group of approximately 15–20 adults. More details about the specific *Texercise Select* intervention components can be found elsewhere[17]. The Texas A&M Institutional Review Board (IRB) approved all study protocol and informed consent was presented in written form to all participants in accordance with these approved protocols (IRB # IRB2015-0024D) and in accordance with the guidelines of the Declaration of Helsinki.

To assess the program effect on physical activity levels and associated costs, we conducted an evaluation of the program spanning 2015–2017 in North, Central, and East Texas. Overall, 9 intervention group sites were included as intervention delivery sites with an average number of 18 participants per intervention delivery site. In addition, 14 comparison sites were included to compare relative changes (if any) in physical activity. Given this is an observational study lacking random assignment, there may be differences in the probability of participating in the intervention (treatment). As further discussed in the Statistical Analysis section that follows, inverse probability of treatment weighted (IPTW) estimation using propensity scores is an important causal inference method widely used in current observational studies and was introduced to estimate the treatment effect in adjusted analyses [18, 19]. Through controlling for covariates that determine and influence participants’ decision to take part in the intervention, inverse probability of treatment weighted estimation or IPTW helps create comparable and representative comparisons between intervention (treatment) and comparisons groups [18]. This method accounts for confounding issues such as nonrandom program participation and differences in observed characteristics of subjects, and further helps to reduce bias[18, 19]. Intervention delivery sites and comparison sites were similar and included senior centers, community centers, faith-based organizations among other similar settings. Only participants at intervention delivery sites were exposed to the intervention, while those at comparison sites were not exposed to the intervention. Sites were selected pragmatically without random assignment, as both intervention delivery sites and comparison sites had to agree (e.g., senior center directors) to participate. The planned implementation of a delayed intervention for comparison sites was meant to lessen self-selection bias into the intervention, as comparison sites would have a later opportunity to participate in the intervention.

### Objective measurement using accelerometers

Physical activity outcomes were measured using objective measures of physical activity using accelerometers (BodyMedia SenseWear[20]) worn on the upper left arm among a subset of the overall subjective survey group (given limited availability of devices). Data collected and used from accelerometers included ‘duration on-body’ in minutes to calculate daily wear time and physical activity minutes categorized into moderate and vigorous using METs or Metabolic Equivalents[21]. This included minutes of moderate-to-vigorous physical activity or MVPA. Individuals who were able to wear the device for at least 10 consecutive days were invited to participate in this subgroup. Data from individuals with daily wear times of at least 10 hours[22] who also wore the devices for at least 7-days within a 10-day period were included in analyses. We excluded any days with less than 10 hours per day of wear time[3, 23]. We used the 7-day threshold of having at least 10 hours of wear time as we wanted to measure one week (7-days) of physical activity. Others have used less conservative thresholds for the number of days per week of wearing the devices (e.g., at least 6 days[24]), thus our use of 7 days is a direct measure of one week rather than using a subset of 7-days and extrapolating to one week. The total sample size used for accelerometer group using objective measures (those who wore accelerometers) included 38 individuals in the intervention group and 55 individuals in the comparison group.

### Subjective measurement using surveys

Paper surveys were provided to participants to complete in person at baseline, immediately following the program, and at the longer-term follow-up. For the purposes of this study, we used a recall of both the number of days one engaged in physical activity during the past week and the average minutes of physical activity on those days. Questions were asked separately for moderate physical activity and vigorous physical activity. These questions were modeled from the International Physical Activity Questionnaire (IPAQ)[25]. Moderate-to-vigorous minutes of physical activity (MVPA) were calculated using a combination of the survey items using a multiplier of 2 for vigorous minutes as described in the Physical Activity Guidelines for Americans (2008)[26]. The recommended dose of MVPA per week is 150 minutes[26]. As such, we used both a measure of MVPA minutes (continuous) and the threshold of 150 minutes of MVPA for analyses of whether individuals were inactive (reporting 0 minutes of MVPA), insufficiently physically active (reporting 1–149 minutes of MVPA), and sufficiently physical activity (reporting 150 or more minutes of MVPA) [27]. The total sample size used for subjective measures (those completing the survey) included 65 individuals in the intervention group and 102 individuals in the comparison group.

### Timeline of measurement

**Fig 1:**
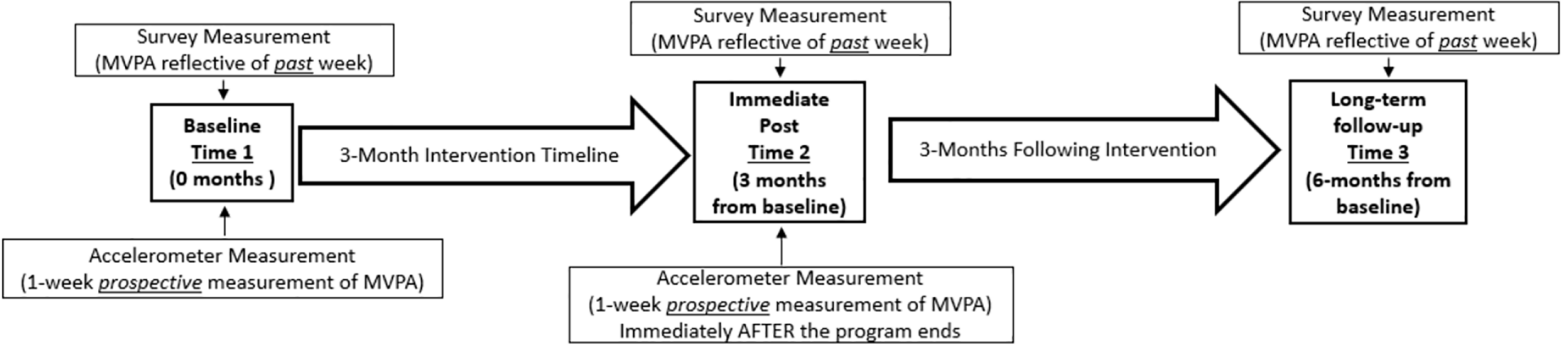
Project timeline and measurement.

As illustrated in Fig 1, multiple measurements were taken for both the intervention group and the comparison group to assess whether and to what extent there were changes over time, and if changes were sustained. There were 3 assessment time points: 1) ***baseline*** defined as the time immediately prior to intervention exposure; 2) ***immediate post*** which is equivalent to approximately 3-months from baseline where survey measurement was conducted immediately after the last session of the program (surveys asked subjective MVPA referring to the *previous* week (during the intervention), while accelerometers were tracked *immediately following* the end of the program); and 3) ***long-term follow-up*** equivalent to approximately 3 months from the close of the program, which is also equivalent to approximately 6-months from baseline. We ran analyses using the survey measures only among the baseline and 6-month time points given the timeline for measurement for the accelerometers at ***immediate post*** was after the program, whereas the measure of subjective MVPA at ***immediate post*** was inclusive of the past week (during the program implementation and before wearing the accelerometers). As such, we did not use subjective measures for ***immediate post***, given the non-overlapping measurement timelines.

### Statistical analyses

SAS 9.4 (Cary, NC) was used for all statistical analyses. To test for significant differences in demographics across intervention and comparison group status were employed bivariate logistic regression. Paired t-test were used to present bivariate comparisons for the accelerometer group and for bivariate analysis of the survey group. These models were used to test hypotheses related to improvements in physical activity comparing time 1 to time 2 (accelerometer measured weekly minutes of MVPA) and comparing time 1 to time 3 (survey measured weekly minutes of MVPA) (see Fig 1). Linear mixed models (random coefficient models) were used to model MVPA while accounting for time (repeated measures nested within individuals) and individuals nested within site locations incorporating random intercepts among the survey group. These models allowed us to test hypotheses related to both comparisons from time 1 to time 3 (survey measured weekly minutes of MVPA) and comparisons across the intervention and comparison group testing for potential differential effects.

Further, propensity scores using inverse probability of treatment weighted (IPTW) estimation[19] were used.

Propensity score, denoted as *P*(*Z* = 1|*X*), is a probability that indicates the likelihood of participants being enrolled in the program (denoted as Z = 1) conditional on the matching covariates (denoted as a set X) [18]. It accounts for fundamental differences that determine program participation. After controlling for these differences, program participation is considered to be random and participants are comparable regarding health outcomes.

Specifically, the propensity score is estimated with logit regression of treatment participation on matching covariates. Age, sex, race/ethnicity, and chronic conditions have been shown to be associated with intervention completion in prior research[28]. Thus, in our study, the matching covariates are those sociodemographic variables that may influence individuals’ decision to participate in the program, which included age (continuous), sex (male, female), race and ethnicity (non-Hispanic White versus other), comorbidity (having 2 or more chronic conditions versus not), and education (3-level: high school degree or equivalent, some college or technical school, college graduate). Individuals with a higher propensity score have a higher probability to take part in the program and vice versa.

After obtaining the propensity score, there are different ways to utilize it in the analysis, such as regression adjustment, matching, and inverse probability of treatment weights (IPTW) [29]. Different from direct propensity score matching, the IPTW is used as a weight for model estimation and is calculated from propensity score as 1/*P*(*Z* = 1|*X*) for treatment group participants and 1/(1 − *P*(*Z* = 1|*X*)) for control group participants [30, 31]. By means of adding the weights in the model, we created weighted treatment and control groups that are comparable in terms of individual characteristics. More importantly, program participation is orthogonal to individual characteristics, which also means the confounding factors leading to bias are correctly controlled. To estimate MVPA in multi-level models (random coefficient models with random intercepts) with repeated measures, we employed a fully adjusted model adding the IPTW in the model.

### Medical cost savings related to physical activity among the survey group

To obtain an estimate of potential cost savings from the intervention program (i.e., only exploring the intervention group), we make use of the cost estimates associated with physical activity level change in Carlson, S.A., et al. (2015) [27]. In their study, the authors merged the National Health Interview Survey (2004–2010) with the Medical Expenditure Panel Survey (2006–2011). They applied the generalized linear model to estimate the health expenditure associated with level change from inactive or insufficiently physically activity to sufficiently physically active, respectively. The relative difference from baseline to 6 months calculated for each individual in the intervention subjective survey group separately (i.e., following person ‘X’ from baseline to 6 months). For example, if person ‘X’ was *inactive* at baseline and moved to being *insufficiently active* at 6 months that would be associated with a gain or savings in medical costs, so too if person ‘X’ moved from *insufficiently physically active* to *sufficiently physically active* and so too from *inactive* to *sufficiently physically active* and vice versa in the negative direction.

Using these estimates, we can quantify physical activity changes in our study as the health expenditure saved. Thus, this allowed for testing our hypothesis of gains in physical activity associated with medical costs savings. More specifically, the annual health expenditure of inactive people relative to active people per capita is $1437 and annual health expenditure of insufficient active people relative to active people per capita is $713. These health expenditures were adjusted to 2012 dollars according to the Personal Health Care Expenditure Price Index (hereinafter referred to as PHCEPI)[32]. Accordingly, we converted our cost saving estimated from physical activity changes in the repeated measures random coefficient model to 2015 dollars using the PHCEPI given that 2015 price index is the most updated index available from the Medical Expenditure Panel Survey. We also used the Consumer Price Index for All Urban Consumers: Medical Care (hereinafter referred to as CPIMC) which contains 2017 index to convert our cost estimates to 2017 dollars. In the results section, we report both cost estimates using the 2015 PHCEPI and 2017 CPIMC. Further, the medical cost is also compared to the relative program cost, estimated to be $229 per participant using multiple measures (e.g., estimated time volunteered for facilitators, supplies), with more detail reported elsewhere[33].

## Results

### Demographics

#### Accelerometer subgroup

The accelerometer groups (intervention, n = 38; comparison, n = 55) were comparable for key characteristics related to physical activity including: mean age (within 3 years on average, but with a significantly lower mean age (p = 0.0383) in the intervention group) at 72 years (range 60–88 years) and 75 years (range 62–93 years); sex (at 90% and 76% female); those with comorbidities at 65% and 70%; and education (at 45% and 32% who were high school graduates or who had less than high school) for the intervention and comparison group, respectively (see Table 1). The groups were somewhat less similar in terms of race and ethnicity (significantly different at p = 0.0012; with 74% and 62% non-Hispanic White) for the intervention and comparison group, respectively.

**Table 1:** Demographic characteristics of the analytical sample of objective (accelerometer) measurement at baseline.

		Intervention among matched analytical sample				Comparison			
		All (n = 38)		<150 minutes of weekly MVPA at baseline (n = 27)		All (n = 55)		<150 minutes of weekly MVPA at baseline (n = 42)	
		Mean (range)	Standard deviation	Mean (range)	Standard deviation	Mean (range)	Standard deviation	Mean (range)	Standard deviation
**Age**	Years	72.00 (60–88)	6.81	72.22 (60–88)	7.62	75.13 (62–93)	7.50	74.98 (62–93)	7.99
		**N**	**Percent**			**N**	**Percent**		
**Sex**	Female	34	89.47	25	92.59	42	76.36	34	80.95
	Male	4	10.53	2	7.41	13	23.64	8	19.05
**Race**	Minority	9	25.71	8	33.33	21	38.18	25	59.52
	Non-Hispanic White	26	74.29	16	66.67	34	61.82	17	40.48
**Comorbidity**	Comorbidities	24	64.86	17	65.38	38	70.37	33	80.49
	No comorbidities	13	35.14	9	34.62	16	29.63	8	19.51
**Education**	High school graduate or less than high school	17	44.74	11	40.74	17	31.48	11	26.83
	Some college or vocational school	14	36.84	10	37.04	15	27.78	13	31.71
	College graduate	7	18.42	6	22.22	22	40.74	17	41.46

Demographic Comparisons: Bivariate logistic regression predicting group status (intervention-comparison) identified differences (p < .05) between case and control included: among all across race (p = 0.0012) and age (p = 0.0383); Among insufficiently active at baseline across race (p = 0.0438).

Those failing to meet the MVPA guidelines at baseline (intervention, n = 27; comparison, n = 42) were also comparable for a key characteristic related to physical activity including mean age 72 years (range 60–88) and 75 years (range 62–93 years); sex (at 93% and 81% female); those with comorbidities (at 65% and 81%); and education (at 37% and 32% with some college or vocational school) for the intervention and comparison group, respectively. The groups were somewhat less similar in terms of race and ethnicity (significantly different at p = 0.0438; 67% and 41% non-Hispanic White) for the intervention and comparison group, respectively.

#### Survey measurement

The survey groups (intervention, n = 65; comparison, n = 102) were comparable for key characteristics related to physical activity including: mean age 74 years (range 61–91 years) and 75 years (range 58–95 years); sex (80% and 77% female); those with comorbidities at 75% and 71%; and education (at 28% and 25% with a college or technical degree) for the intervention and comparison group, respectively (see Table 2). Race and ethnicity differed (significantly different at p<0.0001; with 63% and 36% non-Hispanic White) for the intervention and comparison group, respectively.

**Table 2:** Demographic characteristics of the analytical sample of subjective (survey) measurement at baseline.

		Intervention among matched analytical sample (n = 65)				Comparison (n = 102)			
		All (n = 65)		<150 minutes of weekly MVPA at baseline (n = 48)		All (n = 102)		<150 minutes of weekly MVPA at baseline (n = 78)	
		Mean (range)	Standard deviation	Mean (range)	Standard deviation	Mean (range)	Standard deviation	Mean (range)	Standard deviation
**Age**	Years	73.45 (61–91)	7.60	73.19 (62–91)	7.79	75.39 (58–95)	7.89	75.63 (58–95)	8.09
		**N**	**Percent**			**N**	**Percent**		
**Sex**	Female	52	80.00	41	85.42	78	76.47	61	78.21
	Male	13	20.00	7	14.58	24	23.53	17	21.79
**Race**	Minority	23	37.10	13	28.89	63	63.64	47	61.04
	Non-Hispanic White	39	62.90	32	71.11	36	36.36	30	38.96
**Comorbidity**	Comorbidities	49	75.38	35	72.92	69	71.13	55	73.33
	No comorbidities	16	24.62	13	27.08	28	28.87	20	26.67
**Education**	High school graduate or less than high school	21	32.31	14	29.17	47	46.53	34	44.16
	Some college or vocational school	26	40.00	21	43.75	29	28.71	23	29.87
	College graduate	18	27.69	13	27.08	25	24.75	20	25.97

Note: Sample size may vary by up to 3 observations for missing data for those in the intervention and up to 5 observations for those in the comparison.

Demographic Comparisons: Bivariate logistic regression predicting group status (intervention-comparison) identified differences (p < .05) between case and control included: among all across race and ethnicity (p < .0001); Among insufficiently active at baseline across race and ethnicity (p < .0001).

Those failing to meet the MVPA guidelines at baseline were also comparable for key characteristics related to physical activity including: mean age 73 years (range 62–91 years) and 76 years (range 58–95 years); sex (at 85% and 78% female); education (at 27% and 26% with a college or technical degree); and having comorbidities (at 73% and 73%) for the intervention and comparison group, respectively. Race and ethnicity varied (significantly different at p<0.0001; with 71% and 39% non-Hispanic White) for the intervention and comparison group, respectively.

### Bivariate comparisons

#### Accelerometer subgroup

**Fig 2:**
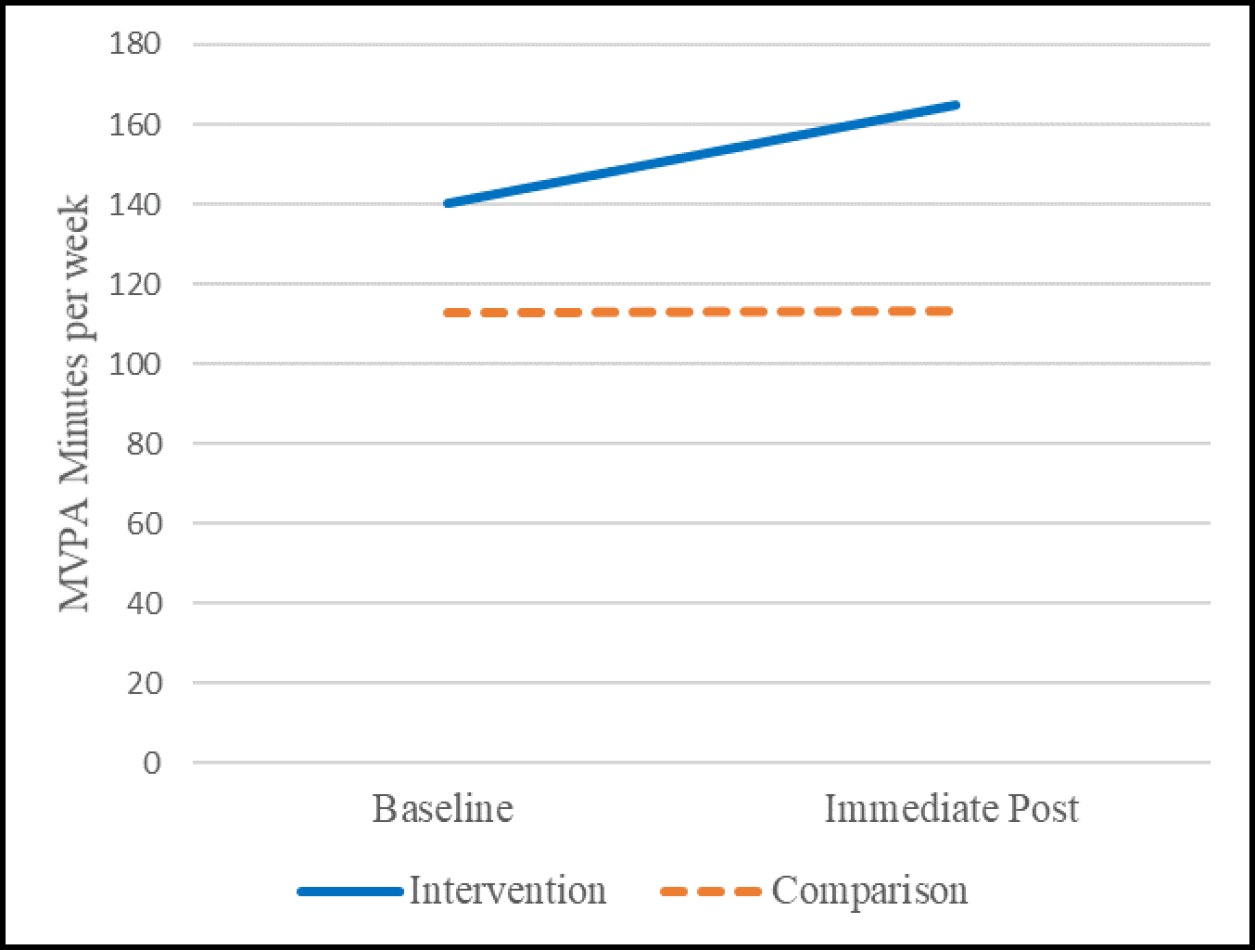
Unadjusted differences among the overall sample using accelerometers.

Case: Among the intervention group assessed with accelerometers (n = 38), overall PA average weekly minutes of MVPA were at 140 (range 0–1144) and 165 (range 0–920) for baseline and immediate post intervention, respectively (see Table 3). Among the insufficiently active population at baseline (i.e., those not achieving 150 minutes of MVPA, n = 27) the weekly minutes were 51 (range 0–146) and 90 (range 0–329) at baseline and immediate post, respectively. There was no pre/post difference (paired n = 38, p = 0.3943) overall; however, there was a significant improvement (paired n = 27, p = 0.0210) in MVPA among those insufficiently active at baseline with an average mean difference of 39 minutes (see Table 4). Fig 2 presents the unadjusted means at baseline and immediate post.

**Table 3:** Mean weekly MVPA among the objective measurement group at baseline and immediate post.

	Intervention				Comparison			
	All (n = 38)		<150 minutes of weekly MVPA at baseline (n = 27)		All (n = 55)		<150 minutes of weekly MVPA at baseline (n = 42)	
	Mean (range)	Standard deviation	Mean (range)	Standard deviation	Mean (range)	Standard deviation	Mean (range)	Standard deviation
Baseline weekly MVPA minutes	140.29 (0–1144)	217.36	51.22 (0–146)	53.23	113.22 (0–898)	171.47	36.10 (0–133)	37.70
Post weekly MVPA minutes	165.03 (0–920)	204.15	89.70 (0–329)	98.22	113.31 (0–1382)	239.75	52.74 (0–454)	93.46

**Table 4:** Mean weekly MVPA and associated differences from baseline to immediate post among the objective measurement group.

	Intervention						Comparison					
	All (n = 38)			<150 minutes of weekly MVPA at baseline (n = 27)			All (n = 55)			<150 minutes of weekly MVPA at baseline (n = 42)		
	Mean change	Standard deviation	p-value	Mean change	Standard deviation	p-value	Mean change	Standard deviation	p-value	Mean change	Standard deviation	p-value
Weekly MVPA minutes	24.74	176.9	0.3943	38.48	81.4037	0.0210	0.09	155.7	0.9966	16.64	80.11	0.1856
Effect size	0.1399			0.4727			0.0006			0.2077		

**Fig 3:**
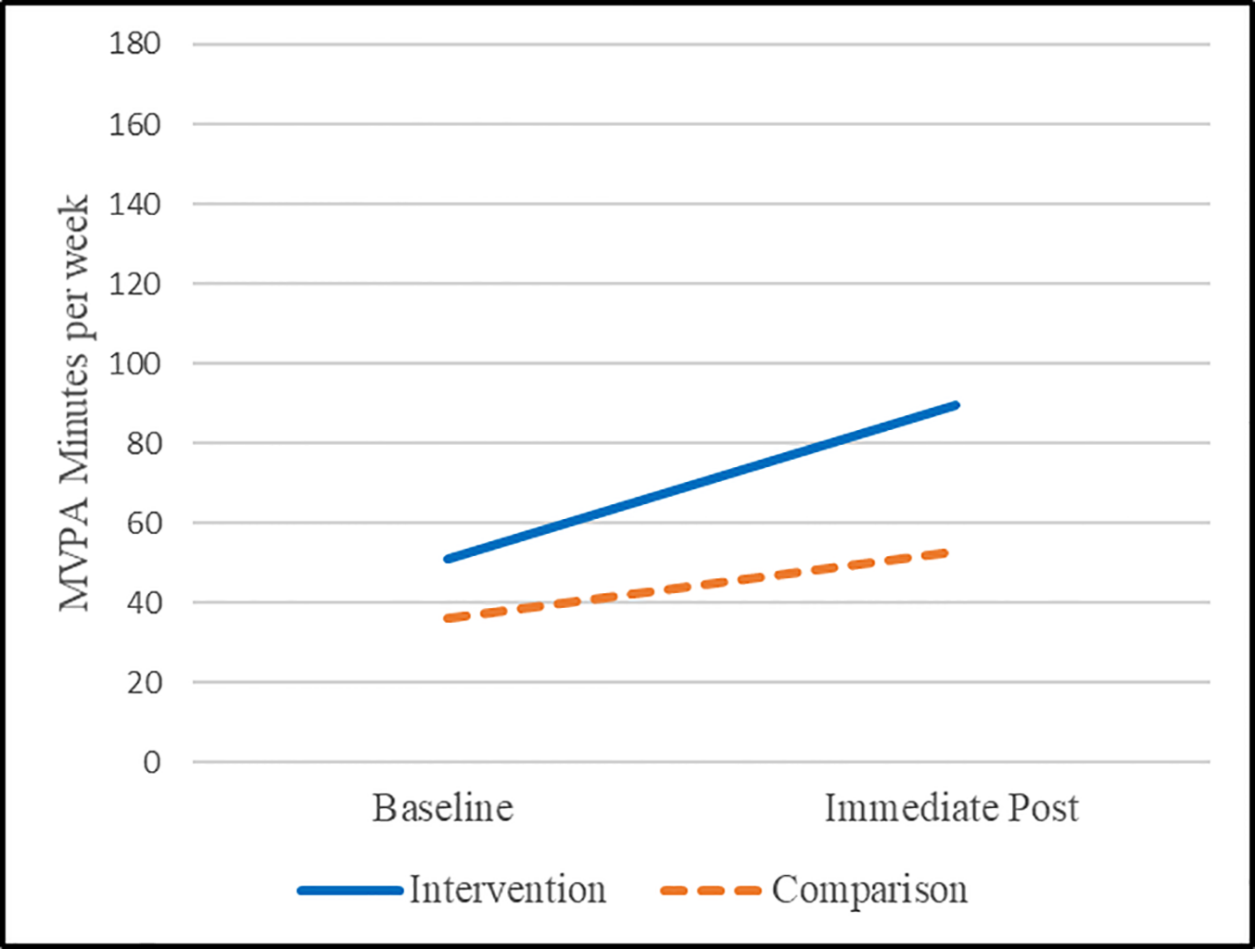
Unadjusted differences among insufficiently active (at baseline) individuals using accelerometers.

Comparison: When assessing the comparison group (not exposed to the intervention), overall PA average weekly minutes of MVPA were at 113 (range 0–898) and 113 (range 0–1382) among all (n = 55) and at 36 (range 0–133) and 53 (range 0–454) among those insufficiently active at baseline (n = 42) (see Table 3). There were no differences among the overall group (paired n = 55, p = 0.9966) or the insufficiently active at baseline group (paired n = 42, p = 0.1856) as compared to immediate post (see Table 4). Fig 3 presents the unadjusted means at baseline and immediate post among those who were insufficiently active at baseline. Fig 3 demonstrates the improvement among those insufficiently active at baseline among the intervention group showing significant improvements in MVPA over baseline with a moderate effect size (paired n = 27, p = 0.0210, effect size: 0.4727) as compared to the comparison group that did not experience significant differences in MVPA over baseline (paired n = 42, p = 0.1856).

#### Subjective assessment

**Fig 4:**
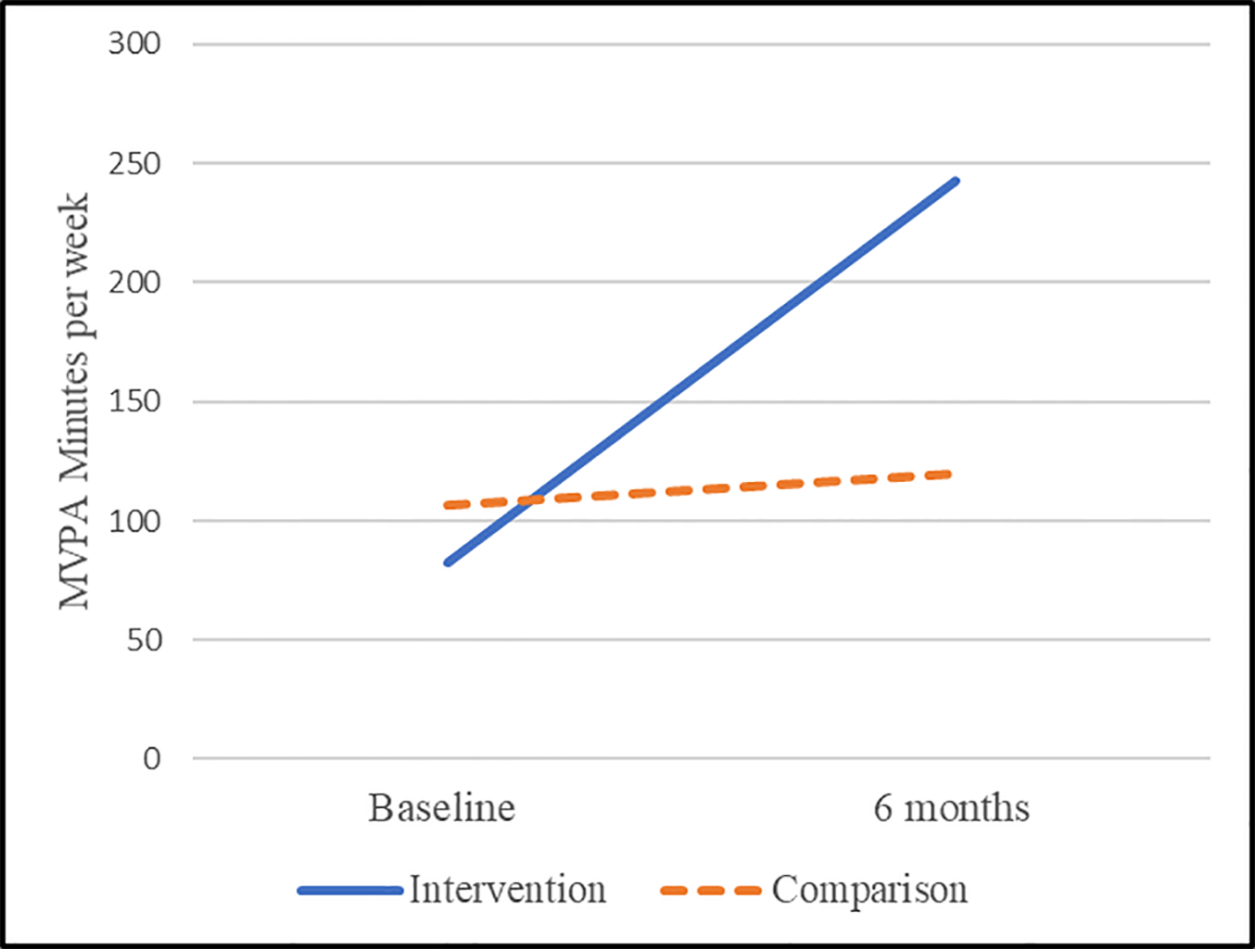
Adjusted differences among the overall sample using propensity score adjustment.

**Fig 5:**
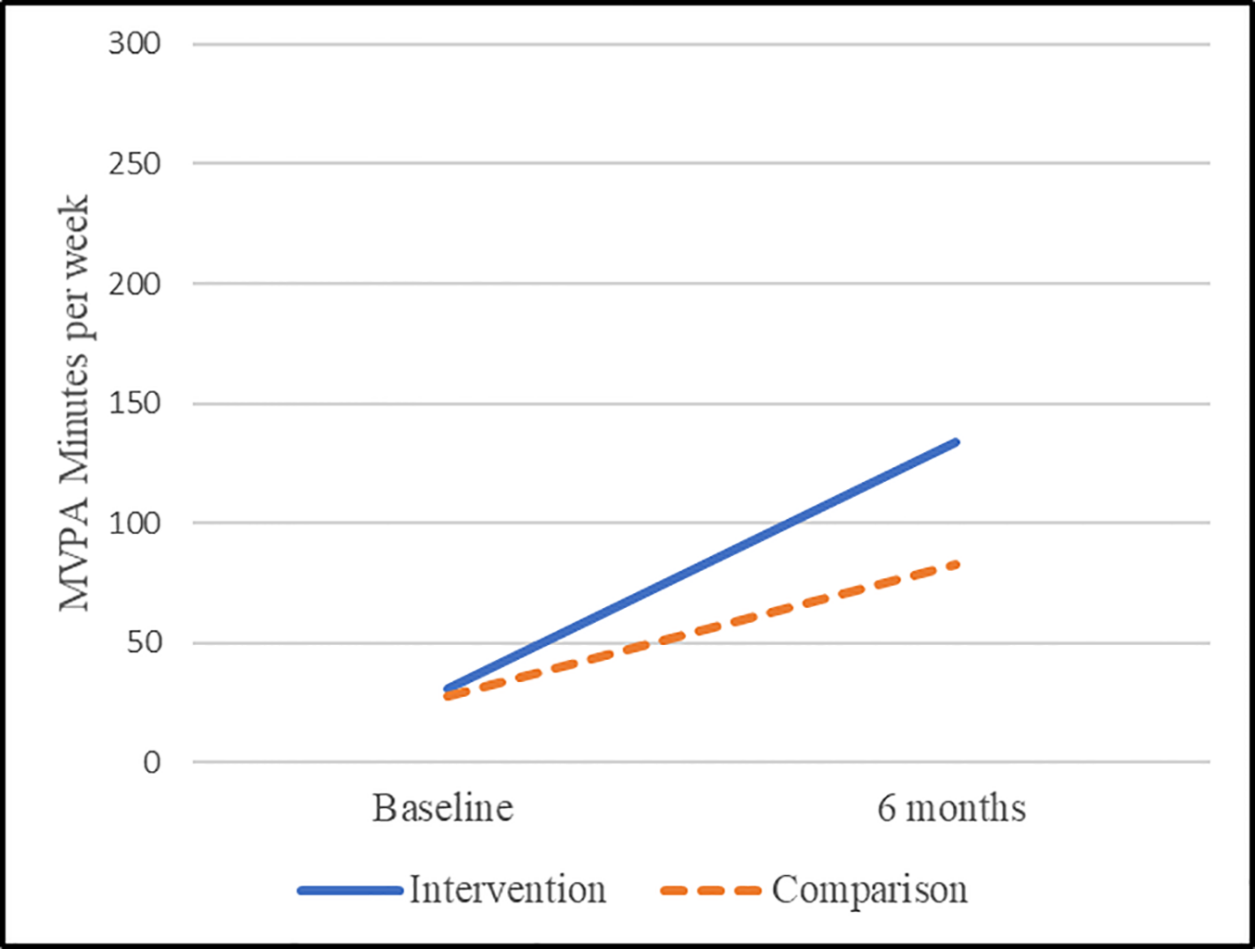
Adjusted differences among insufficiently active (at baseline) individuals using propensity score adjustment.

*Case*: Among the intervention group assessed with surveys including those with 6-month data matched on pre/post time points (n = 65 paired pre/6-months), overall PA average weekly minutes of MVPA were at 87 (range 0–540) and 182 (range 0–3780), respectively (see Table 5). Among those insufficiently active at baseline including those with 6-month data matched on pre/post time points (n = 48 paired pre/6 months), overall PA average weekly minutes of MVPA were at 30 (range 0–140) and 125 (range 0–720) for baseline and 6-month assessment, respectively. There was no pre/post difference (paired n = 65, p = .0934) among the intervention; however, there was a significant improvement (paired n = 48, p = 0.0002) in MVPA among those insufficiently active at baseline with a mean difference of 94 minutes with a moderate effect size (effect size: 0.5945) (see Table 6 and Figs 4 and 5).

**Table 5:** Mean weekly MVPA among the subjective (survey) measurement group at baseline and 6-months.

	Intervention (n = 65)				Comparison (n = 102)			
	All (n = 65)		<150 minutes of weekly MVPA at baseline (n = 48)		All (n = 102)		<150 minutes of weekly MVPA at baseline (n = 78)	
	Mean (range)	Standard deviation	Mean (range)	Standard deviation	Mean (range)	Standard deviation	Mean (range)	Standard deviation
Baseline MVPA minutes	86.74 (0–540)	112.89	30.38 (0–140)	38.50	108.59 (0–1356)	200.02	29.17 (0–135)	37.21
Post MVPA minutes	182.14 (0–3780)	478.53	124.67 (0–720)	160.49	122.13 (0–1260)	195.79	79.23 (0–525)	103.41

**Table 6:** Mean weekly MVPA and associated differences from baseline to 6-months among the subjective (survey) measurement group.

	Intervention						Comparison					
	All (n = 65)			<150 minutes of weekly MVPA at baseline (n = 48)			All (n = 102)			<150 minutes of weekly MVPA at baseline (n = 78)		
	Mean change	Standard deviation	p-value	Mean change	Standard deviation	p-value	Mean change	Standard deviation	p-value	Mean change	Standard deviation	p-value
MVPA minutes	95.4000	451.6	0.0934	94.2917	158.6	0.0002	-13.5392	235.7	0.5631	-50.0641	102.0	< .0001
Effect size	0.2112			0.5945			0.0574			0.4908		

*Comparison*: When assessing the comparison group, overall PA average weekly minutes of MVPA (n = 102 paired pre/6 months), overall PA average weekly minutes of MVPA were at 109 (range 0–1356) and 122 (range 0–1260) for baseline 6 months, respectively (see Table 5). Among the inadequately active population at baseline (i.e., those not achieving 150 minutes of MVPA, n = 78) the weekly minutes were 29 (range 0–135) and 79 (range 0–525) at baseline and 6 months, respectively. There was no overall pre/6-month difference (paired n = 102, p = 0.5631), however there was a significant improvement (paired n = 78, p = <0.0001) in MVPA among those insufficiently active at baseline with a mean difference of 50 minutes (see Table 6 and Figs 4 and 5).

### Adjusted analyses of the subjective survey group

#### Overall sample

*Propensity score model*: Overall, there was an improvement (p<0.0001) in minutes of MVPA of 160 minutes among the intervention from baseline to 6 months (see Table 7). By comparison there was no improvement (p = 0.6267) from baseline to 6 months among the comparison group. Fig 4 presents the adjusted means at baseline and 6 months. Here we see that the comparison group remained relatively stable (p = 0.6267) from baseline to 6 months, while the intervention group experienced a significant improvement over baseline (p<0.0001) with an overall differential effect of the intervention (differences in baseline to 6-months among intervention group and comparison group p = 0.0007; overall interaction term p = 0.0012).

**Table 7:** Adjusted mean weekly MVPA and comparisons from baseline to 6-months among the subjective (survey) measurement group.

				Adjusted mean minutes of MVPA per week	Adjusted difference for Baseline versus 6-months	p-value for comparison	p-value for comparison	p-value for type 3 tests of fixed effects for the overall interaction term
Propensity score adjustment	All	Intervention	Baseline	82.4806	160.62	< .0001	0.0007	0.0012
			6-months	243.10				
		Comparison	Baseline	106.75	13.2149	0.6267		
			6-months	119.97				
	<150 minutes of MVPA per week at baseline	Intervention	Baseline	30.9846	103.02	< .0001	0.0224	< .0001
			6-months	134.01				
		Comparison	Baseline	27.8658	54.7486	< .0001		
			6-months	82.6144				

Fully adjusted models using weighted propensity score predicting MVPA with case/control status and time (time 1, time 2, time 3) and the interaction of case/control status and time among: Model 1) all participants and with an intraclass correlation coefficient (ICC) of 0.2738 or 27.38% calculated with the null model; Model 2) the insufficiently active at baseline group (defined as <150 MVPA minutes per week) with an ICC of 0.1545 or 15.45% calculated from the null model

#### Insufficiently active at baseline sample

*Propensity score model*: Among those insufficiently active at baseline, we find an overall improvement of 103 minutes of MVPA from baseline to 6 months (p<0.0001). By comparison there was an improvement among the comparison group of 55 minutes (p<0.0001), however this improvement was approximately half that of the intervention group (p = 0.0224) indicating an overall differential effect (greater improvement among the intervention) for the intervention relative to the comparison group. Fig 5 presents the adjusted means at baseline and 6 months among those who were insufficiently active at baseline. Here we see that the comparison group experienced a significant improvement over baseline (p<0.0001); while the intervention group also experienced a significant improvement over baseline (p<0.0001) with an overall differential effect of the intervention (differences in baseline to 6 months among intervention group and comparison group p = 0.0224; overall interaction term p<0.0001).

### Medical cost comparison among insufficiently active or inactivity relative to sufficiently active

As indicated in the Methods, there were three groups created based on MVPA categories: 1) inactive (reporting 0 minutes of MVPA); 2) insufficiently physically active (reporting 1–149 minutes of MVPA); and 3) sufficiently physical activity (reporting 150 or more minutes of MVPA). What follows is the relative difference from baseline to 6-months calculated for each individual in the intervention subjective survey group separately (i.e., following person ‘A’ from baseline to 6 months).

#### Overall 6-month matched sample

**Fig 6:**
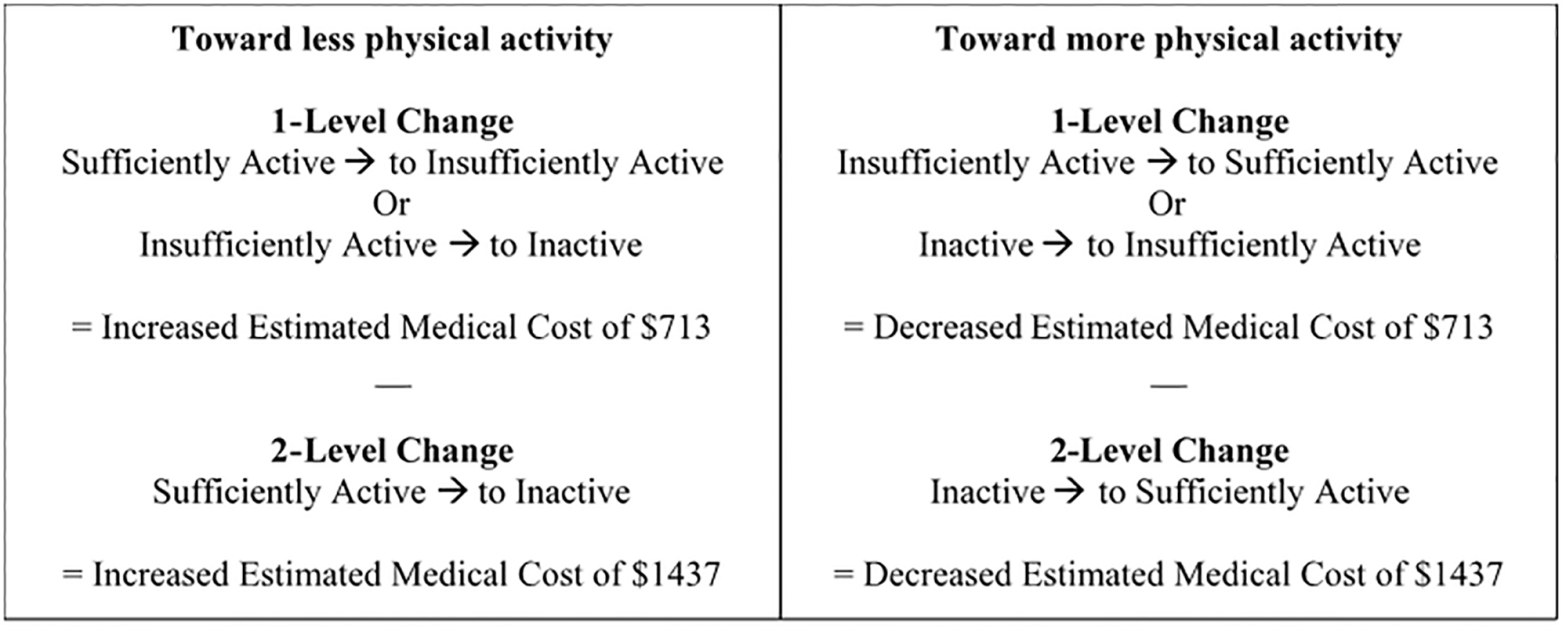
Estimated medical cost differences by reported change in physical activity using estimates from Carlson, S.A., et al. (2015) [27].

The downward movement to a lower physical activity category by *1-level* was associated with a relative cost of $713, while the opposite was associated with a gain of $713 (see Fig 6). Overall, 13 individuals regressed to a lower level of physical activity (e.g., went from insufficiently active to inactive or sufficiently active to insufficiently active). At the same time, 18 individuals gained a level (e.g., went from inactive to insufficiently active or from insufficiently active to sufficiently active) for a net gain of 5 levels at $713 or a total of $3565. At the same time, $3565 in 2012 dollars is adjusted to $3701.73 in 2015 dollars using PHCEPI and $4074.63 in 2017 dollars using CPIMC.

The downward movement from sufficiently activate to inactive would be a change of *2-levels* associated with a relative cost of $1437, while the opposite was associated with a gain of $1437 (see Fig 6). Overall, 3 individuals experience a downward movement by 2 levels from sufficiently active to inactive, while 6 individuals gained 2 levels from inactive to sufficiently active for a net gain of 3 at $1437 or a total of $4311. The $4311 in 2012 dollars is adjusted to $4476.34 in 2015 dollars using PHCEPI and $4927.28 in 2017 dollars using CPIMC. Thereby, among all individuals there was an overall gain of $7876 ($3565 + $4311). Thus, an overall gain of $7876 is adjusted to $8178.07 in 2015 dollars using PHCEPI and $9001.91 in 2017 dollars using CPIMC. While the latest estimates of the cost per participant (n = 65) for the intervention was $229[33]. Thereby, there was an overall *cost* of $7009 (-$14885 +$7876), yet after adjusting to 2015 dollars the cost was $6706.93 (-$14885 + $8178.07) and $5883.09 (-$14885 + $9001.91) using 2017 CPIMC. The mean cost of $107.83 per participant ($7009 / 65 participants), which is lower than the initial cost of $229 per participant given the overall estimated medical cost savings achieved through gains in MVPA; or a mean cost of $111.97 in 2015 dollars using PHCEPI and $123.25 in 2017 dollars using CPIMC per participant.

#### 6-month matched sample among those insufficiently active at baseline

The downward movement to a lower physical activity category by *1-level* was associated with a relative cost of $713, while the opposite was associated with a gain of $713. Overall, 5 individuals regressed to a lower level of physical activity (e.g., went from insufficiently active to inactive). At the same time, 18 individuals gained a level (e.g., went from inactive to insufficiently active or from insufficiently active to sufficiently active) for a net gain of 13 levels at $713 or a total of $9269. At the same time, $9269 in 2012 dollars is adjusted to $9624.50 in 2015 dollars using PHCEPI and $10594.04 in 2017 dollars using CPIMC.

The gain from inactive to sufficiently activate would be a change of *2-levels* associated with a relative gain of $1437. Overall, 6 individuals gained 2 levels from inactive to sufficiently active for a net gain of 6 at $1437 or a total of $8622. Thereby, an overall gain of $8622 is adjusted to $8952.68 in 2015 dollars and $9854.55 in 2017 dollars using CPIMC.

Thus, among insufficiently active at baseline, there was an overall gain of $17891 ($9269 + $8622) yet after adjusting to 2015 dollars the cost saving (gain) was $17891 ($9269 + $8622) was adjusted $18577.18 in 2015 dollars using PHCEPI and $20448.59 in 2017 dollars using CPIMC. Given the latest estimates of the cost per participant (n = 48) was $229. Thereby, there was an overall gain of $6899 (-$10992 + $17891). The overall gain of $6899 (-$10992 + $17891) was adjusted $7585.18 (-$10992 + $18577.18) in 2015 dollars using PHCEPI and $9456.59 (-$10992 + $20448.59) in 2017 dollars using CPIMC. The mean cost savings (gain) of $143.73 per participant *over* the cost of $229 or a mean cost savings (gain) of $149.24 in 2015 dollars using PHCEPI and $164.28 in 2017 dollars using CPIMC per participant *over* the cost of $229.

## Discussion

Overall, this lifestyle intervention program was successful at improving physical activity over and above the comparison group, especially among those insufficiently active at baseline, confirming our study hypotheses. When assessing immediate MVPA changes following the intervention using objective measurement, we found significant gains at 39 minutes of weekly MVPA among insufficiently active individuals with no gains among the comparison group. This indicates targeting the intervention to those not already engaging in adequate levels of MVPA may carry the most benefit and is recommended as an important consideration for those in the aging services sector seeking similar gains in MVPA.

*Texercise Select* is not the only physical activity-related intervention targeting middle-aged and older adults. However, multiple characteristics of *Texercise Select* can be attractive[34]. For example, the physical activity components within the sessions are purposefully meant to be adaptive to both those with less mobility and those with greater mobility. This is accomplished by having multiple types of physical activity presented in the sessions with the participant deciding on their preferred physical activity (e.g., sitting/chair-based activities, activities that can be conducted standing while holding the back of a chair for support with stability, or standing independent of any support). That said, one limitation of this approach might be that the program may be less attractive to those individuals that prefer more intense physical activity (e.g., vigorously intense cardio activity). Another limitation of *Texercise Select* is that some may not prefer the group-based nature of the program and may prefer independent physical activity[35]. Other interventions targeting physical activity (e.g., EnhanceFitness) have found gains in strength, balance, and increased physical activity[36]. Those seeking to implement similar interventions should be able to choose from multiple interventions depending on their constituents (e.g., target population), training infrastructure (a professional versus lay-led model), and available resources (e.g., consideration of cost-benefit ratios). The menu of programs available to middle-aged and older adults varies by geography across the nation and may be lacking particularly in at-risk areas (e.g., rural areas)[37]. *Texercise Select* is recommended to promote sustained gains in MVPA, especially among less physically active individuals. The potential generalizability of our findings is substantial given that less than 45% of older Americans are meeting the Surgeon General’s recommended physical activity levels[38–40].

Individuals who may not be meeting the MVPA guidelines of 150/75 minutes per week are more at risk of adverse health outcomes (e.g., falls[41]) due to insufficient physical activity. As such, these individuals are a critical population to target for intervention in order to ameliorate preventable adverse health outcomes that may be associated with significant costs (e.g., falls)[42]. Given physical activity is not the only issue facing older adults, multiple interventions are necessary to target overall improvements in health (e.g., chronic disease[3], falls[4], nutrition[6]). As a multimodal intervention strategy addressing physical activity and other factors (e.g., nutrition, goal planning) highly associated with the onset and progression of chronic diseases and other related geriatric conditions, *Texercise Select* may be especially appropriate for addressing outcomes other than physical activity.

In addition, behavioral and exercise-intensive health interventions, such as *Texercise Select*, are not recommended as sufficient in improving physical activity alone. Multilevel interventions (e.g., social support, walkable environments)[43] may also hold promise in achieving sustainable gains in MVPA. For example, the built environment (e.g. activity-friendly community design) is a critical component in affecting physical activity among middle-aged and older adults[44, 45] and as such *Texercise Select*, as well as other interventions with similarly impressive effects on MVPA are recommended as a complementary solution to improving physical activity among the nation’s growing middle aged and older adult population. Thus, we recommend that *Texercise Select* be implemented in conjunction with multi-factorial interventions.

When assessing longer-term sustained MVPA using subjective measures, we found that those in the intervention group (both overall and in the insufficiently active group) improved substantially with 160 and 103 minutes of weekly MVPA, respectively. When assessing overall gains in estimated medical cost savings, we found that those who were insufficiently active at baseline achieved a relative cost savings from baseline to 6 months over and above the estimated cost of the intervention. As such, the relative investment in delivery of the program was met with a net savings if delivered to insufficiently active adults. The relative cost savings associated with physical activity in the US[27] is consistent with data from the United Kingdom (UK) showing a difference in cost associated with transitioning from sedentary to active (operationally defined as meeting the PA guidelines of 150/75 minutes of MVPA per week)[46]. Thus, evidence suggests that the medical cost associated with inadequate physical activity can be transformed into savings through sustained gains in MVPA associated with participation in *Texercise Select*.

### Limitations

Several limitations should be taken into consideration when evaluating the implications of this study. While the design (case/comparison) was more rigorous than a simple pre/post design, the results may not be generalizable to larger populations, given the relatively small sample size. Given MVPA has not been evaluated previously with this program, we did not conduct power analyses on expected changes in MVPA for this study, and as such took a pragmatic approach to obtain a minimum of 100 intervention participants and 100 comparison participants with at least two time points of data (i.e., pre and immediate post data). Of note, data collection on multiple sites was disrupted (no data was collected) due to Hurricane Harvey (2017) in and around the greater Houston area preventing six-month data collection for multiple sites. In addition, the use of self-reported subjective survey data for the overall sample may be subjected to recall bias of participants. Relying on self-reported survey data (e.g., among the 6-month comparison) was a major limitation, as ideally objectively measured data (e.g., using accelerometers) would help avoid the potential for either over or underreporting physical activity and provide more reliable and valid data. Further, this study lacked physiological or functional data supporting the effectiveness of the intervention. Evidence suggests using accelerometers provides highly accurate records of physical activity[47, 48], which is a major strength. Including objective measures of physical activity was a substantial strength in subgroup analyses from baseline to immediate post. Further, the intervention group was different than the comparison group across a variety of characteristics (e.g., race and ethnicity). This is a major limitation, yet the two groups were matched on a major characteristic related to physical activity, which is age. The extent to which the results of this specific intervention can be generalized to other physical activity interventions is not clear. Further research may explore comparisons across different interventions targeted to this population. The use of propensity scores was a major strength to counter this limitation as it works to employ causal inference methods to remove bias introduced by nonrandom program designs. Moreover, we did not have direct measures of medical costs associated with transitions (e.g., from inactive to insufficiently activate), but did use an estimated cost from national expenditure data that controlled for age, sex, race/ethnicity, education, marital status, smoking status, poverty level, health insurance status, metropolitan statistical area, and US Census region([27]p. 320).

### Conclusions

The findings in the current study suggest that *Texercise Select* is effective at increasing initial and longer-term MVPA and that the sustained effect among at-risk individuals (insufficiently active individuals who come into the program) carries significant estimated medical cost savings. More extensive research with a larger sample size may lead to even greater insight into potential medical cost savings and ultimately help guide public policy.
